# Added values of DXA-derived visceral adipose tissue to discriminate cardiometabolic risks in pre-pubertal children

**DOI:** 10.1371/journal.pone.0233053

**Published:** 2020-05-13

**Authors:** Li-Wen Lee, Chu-Jung Hsieh, Yun-Hsuan Wu, Yu-San Liao

**Affiliations:** 1 Department of Diagnostic Radiology, Chang Gung Memorial Hospital, Puzi City, Chiayi, Taiwan; 2 Department of Nursing, Chang Gung University of Science and Technology, Puzi City, Chiayi, Taiwan; 3 Department of Diagnostic Radiology, Chang Gung Memorial Hospital, Yunlin, Taiwan; University of Texas Health Science Center at Houston, UNITED STATES

## Abstract

**Background:**

The new generation of dual energy X-ray absorptiometry (DXA) scanners provide visceral adipose tissue (VAT) estimates by applying different algorithms to the conventional DXA-derived fat parameters such as total fat, trunk fat and android fat for the same image data.

**Objective:**

This cross-sectional study aimed to investigate whether VAT estimates from Hologic scanners are better predictors of VAT than conventional DXA parameters in pre-pubertal children, and to explore the discrimination ability of these VAT methods for cardiometabolic risks.

**Methods:**

Healthy pre-pubertal children aged 7–10 years were recruited for basic anthropometric, DXA and magnetic resonance imaging (MRI) measurements. Laboratory tests included lipid profile, glycaemic tests and blood pressure.

**Results:**

All VAT methods had acceptable to excellent performance for the diagnosis of dyslipidaemia (area under the curve [AUC] = 0.753–0.837) and hypertensive risk (AUC = 0.710–0.821) in boys, but suboptimal performance for these risks in girls, except for VAT by MRI in the diagnosis of dyslipidaemia. In both sexes, all VAT methods had no or poor discrimination ability for diabetes risk.

**Conclusions:**

DXA-derived VAT estimates are very highly correlated with standard methods but has equivalent discrimination abilities compared to the existing DXA-derived fat estimates.

## Introduction

Excess fat or obesity is associated with an increased risk of cardiometabolic diseases such as dyslipidaemia, diabetes and cardiovascular disease. In addition to the total fat mass, visceral adipose tissue (VAT) distribution and ectopic fat in non-adipose tissues are regarded as more reliable predictors than total fat mass alone for obesity-related health consequences [1, 2]. The most common approach to assessing adiposity is body mass index (BMI). In children, body adiposity is influenced by sex, age, race and pubertal stage [3, 4]; therefore, a child’s weight status needs to be assessed relative to children of the same age and sex (BMI z-score). BMI and BMI z-score represent indices of excess relative weight rather than fat masses, and there is concern that they may be insufficiently sensitive markers for the evaluation of fat distribution. Anthropometric indices such as waist circumference (WC), hip circumference and sagittal abdominal diameter (SAD) are regarded as better indicators of abdominal fatness. However, these methods are indirect measures of VAT and may not be accurate under some circumstances. Computed tomography (CT) and magnetic resonance imaging (MRI) are standard methods for determining VAT which allow direct measurement of VAT, but they have the disadvantages of high cost and being time-consuming. Dual energy X-ray absorptiometry (DXA) provides total body and regional body composition analysis at lower cost, radiation equivalent to the daily background radiation and short scan time but high precision [5, 6] and for these reasons is a more suitable tool for population screening.

DXA methodology is based on applying two different energies of X-rays to decompose an object into two components with known x-ray attenuation coefficients [7]. With an assumption of the fat fraction of the soft tissue mass in the bone-containing pixels, DXA can further partition the body into lean, fat and bone masses [8]. It is worth noting that the basic methodologies are similar but the algorithms used to partition soft tissues may differ among DXA manufacturers [9, 10]. Conventional DXA also provides cut lines by which to divide the scanned images into the head, trunk, arms and legs, allowing for regional body composition analysis [11]. With the region of interest (ROI) placed in the upper body and low body, DXA can also provide android and gynoid fat estimates, respectively. In DXA images, the android region is defined as the area with its inferior margin at the iliac crest and the superior border defined as 20% of the distance between the iliac crest and the chin [12]. The gynoid region includes the hips and upper thighs, with its superior margin at 1.5 times the android height below the iliac crest and its height two times the height of the android region [12]. Android obesity is known to be more correlated with cardiometabolic risk than gynoid fat deposition [13].

Conventional DXA analysis is based on a 2-dimensional projection image method and cannot be used to differentiate visceral fat from subcutaneous fat in the range of interest. However, the new generation of DXA scanners are equipped with additional VAT assessment options, i.e., Lunar’s CoreScan and Hologic’s InnerCore, that enable automated assessment of the VAT area and volume using specific algorithms. The VAT estimation equations for both DXA manufacturers include similar variables such as the anterior-posterior thickness of the abdomen and the width of subcutaneous layer on lateral abdomen, and the prediction model has been validated against CT-derived VAT in adults [14, 15]. Although VAT_DXA_ volume is measured from a block above iliac crest for both DXA manufacturers, the height of the block varies. The height of the block is 5 cm for the Hologic scanner [14] but 20% of the distance between the iliac crest and the chin (within the android region) for the Lunar scanner [15].

Studies report that metabolic alterations are already detectable in prepubertal boys and girls and the risks are associated with VAT [16–18]. Therefore, validation of VAT methods for early detection and intervention of obesity-related health consequences is appropriate in prepubertal age. VAT_DXA_ has been extensively validated in adults, showing very high correlation with VAT derived from cross-sectional images (r > 0.9) [14, 15, 19]. However, the limited studies in the pediatric population have substantial variation (r = 0.55–0.79) [20, 21], and all were performed with the Lunar scanner. There is high correlation but poor agreement between body composition measures using different DXA systems [22]. Further studies are still needed to investigate the values of VAT_DXA_ estimates provided by the Hologic DXA scanner. This study aimed first to investigate whether VAT_DXA_ estimates in pre-pubertal children from the Hologic scanner are better predictors of VAT than anthropometric indices and the other conventional DXA parameters. Another aim was to explore the discrimination ability of these VAT methods for cardiometabolic risks.

## Methods

### Study design

This cross-sectional prospective study was approved by the Institutional Review Board of the Chang Gung Medical Foundation (No: 101-2952A3) and written informed consent was obtained from all subjects and their parents or guardians after explaining the study procedure. Subjects were recruited via hospital advertisements, word of mouth and outpatient clinic referrals between July 2013 and January 2015.

### Participants

The inclusion criteria were healthy Taiwanese children aged 7–10 years in Tanner stage I. Pubertal stage was assessed by a single physician according to Tanner and Marshall [23]. Children were excluded if they had congenital anomalies, chronic illness, contraindications for an MRI scan or use of regular medication in the last six months. Subjects who presented with a family history of type 2 diabetes, hypertension or gestational diabetes mellitus were also excluded from the study. On the study day, participants presented to an 8:30 am appointment, following an 8- to 12-hour fast. On arrival, participants were asked to void and change into a light hospital gown. All measurements were completed at the same morning session with a total study time of about 2 hours.

### Anthropometric measurement

All anthropometric measurements were carried out by the same investigator throughout the study. Height and weight were measured using a digital scale, with subjects wearing a hospital gown and no shoes. BMI was calculated by dividing weight (kg) by height squared (m^2^). The z-scores for BMI were computed using the WHO AnthroPlus software according to the WHO Reference 2007 [24]. WC was measured at the midpoint between the lowest rib and iliac crest, with the tape parallel to the floor.

### Body composition by DXA

Whole body DXA was performed using a Hologic Delphi A scanner equipped with software version 12.5 (Hologic, Bedford, MA, USA). Then, the obtained DXA images were reanalyzed using Apex version 5.6 (Hologic, Bedford, MA, USA), with the National Health and Nutrition Examination Survey (NHANES) option enabled. Subject position and ROI placement were performed in accordance with the manufacturer’s instructions [25]. Estimates, including total body fat mass, trunk fat mass, android fat mass, total percentage fat (PBF), trunk percentage fat (PBF_TR) and VAT, were generated and compared to VAT obtained by using MRI (VAT_MRI_).

### Body composition by MRI

Breath-hold T1-weighted images of the abdomen and pelvis were acquired on a 3 Tesla Siemens Verio scanner equipped with software syngo MR B17 (Siemens Medical System, Erlangen, Germany). Details of the parameters were as follows: repetition time = 514 ms, echo time = 8.8 ms, field of view = 500×500 mm^2^, matrix = 384×384 and number of excitations = 1. Patients were placed in a supine position and a series of 10–12, 1-cm-thick axial images with a 1 cm gap between images were acquired. VAT_MRI_ volume (cm^3^) was measured between the diaphragm and the pubic symphysis; VAT_MRI_ area (cm^2^) was measured from single-slice MRI at the L4 vertebral level, using image analysis software (SliceOmatic 5.0, TomoVision, Montreal, Quebec, Canada) by the same radiologist. SAD was measured at the level of the L4 vertebral body by measuring the distance from the anterior to the posterior part of the body using ImageJ software [26].

### Outcome definitions

Blood pressure was measured twice on the right arm using an automated sphygmomanometer with an appropriately-sized cuff after the children had rested in the sitting position for five minutes. A third measure was taken if the first two measures differed by ≥ 10 mmHg. The average of the measures was used for further analysis. Blood pressure percentile was categorized on the basis of individual age, sex and height percentile according to the American Academy of Pediatrics Pediatric Hypertensive Guidelines [27]. Children with blood pressure in the < 90^th^ percentile were classified as having normal blood pressure, whereas children with blood pressure in the ≥ 90^th^ percentile were considered at hypertensive risk.

Venous blood samples were collected following body composition measures. Blood glucose and lipid profile were analyzed using a Hitachi 7600 analyzer (Hitachi, Tokyo, Japan) with enzymatic procedure. Glycated hemoglobin (HbA_1_c) values were measured by Premier Hb9210 (Trinity Biotech, Kansas City, MO, USA). Participants with total cholesterol ≥ 170 mg/dl, triglycerides ≥ 75 mg/dl, low-density lipoprotein cholesterol ≥ 110 mg/dl, high-density lipoprotein cholesterol ≤ 45 mg/dl or non-high-density cholesterol ≥ 120 mg/dl were considered at lipid risk [28]. Participants with HbA_1_c ≥ 5.7% or fasting blood glucose ≥ 100 mg/dl were considered at diabetes risk.

### Statistical analysis

All analyses were carried out using SPSS Statistics version 22 (IBM Corp., Armonk, NY, USA). A Student t-test was used to calculate the difference between measurements for both sexes. Pearson’s correlation analysis was used to test the degree of linear relationship between two variables. The Pearson correlation coefficients (r) were classified into perfect (r = 1), very high (0.9 ≤ r < 1), high (0.7 ≤ r < 0.9), moderate (0.5 ≤ r < 0.7) and low (0.3 ≤ r < 0.5). Receiver operating characteristics (ROC) analysis was used to judge how accurately a VAT method could discriminate between children with and without cardiometabolic risk factors. The area under the ROC curve (AUC) was used to quantify the predictive ability of a VAT method as a biomarker for cardiometabolic risk. ROC curves were generated using the same set of subjects for all VAT measurements. The values of the AUC were defined as reflecting acceptable discrimination if 0.7 ≤ AUC < 0.8; excellent discrimination if 0.8 ≤ AUC < 0.9; and outstanding discrimination if AUC ≥ 0.9. MRI was considered as the gold standard for VAT measurements and the other VAT methods were compared to VAT_MRI_. Statistical significance was defined at α = 0.05. Boxplot was plotted for graphically illustration of the data distribution. Heat map was plotted using Microsoft Excel 2016 (Microsoft Corporation, Redmond, WA, USA) to provide a graphical representation of the correlation matrix.

## Results

**Fig 1:**
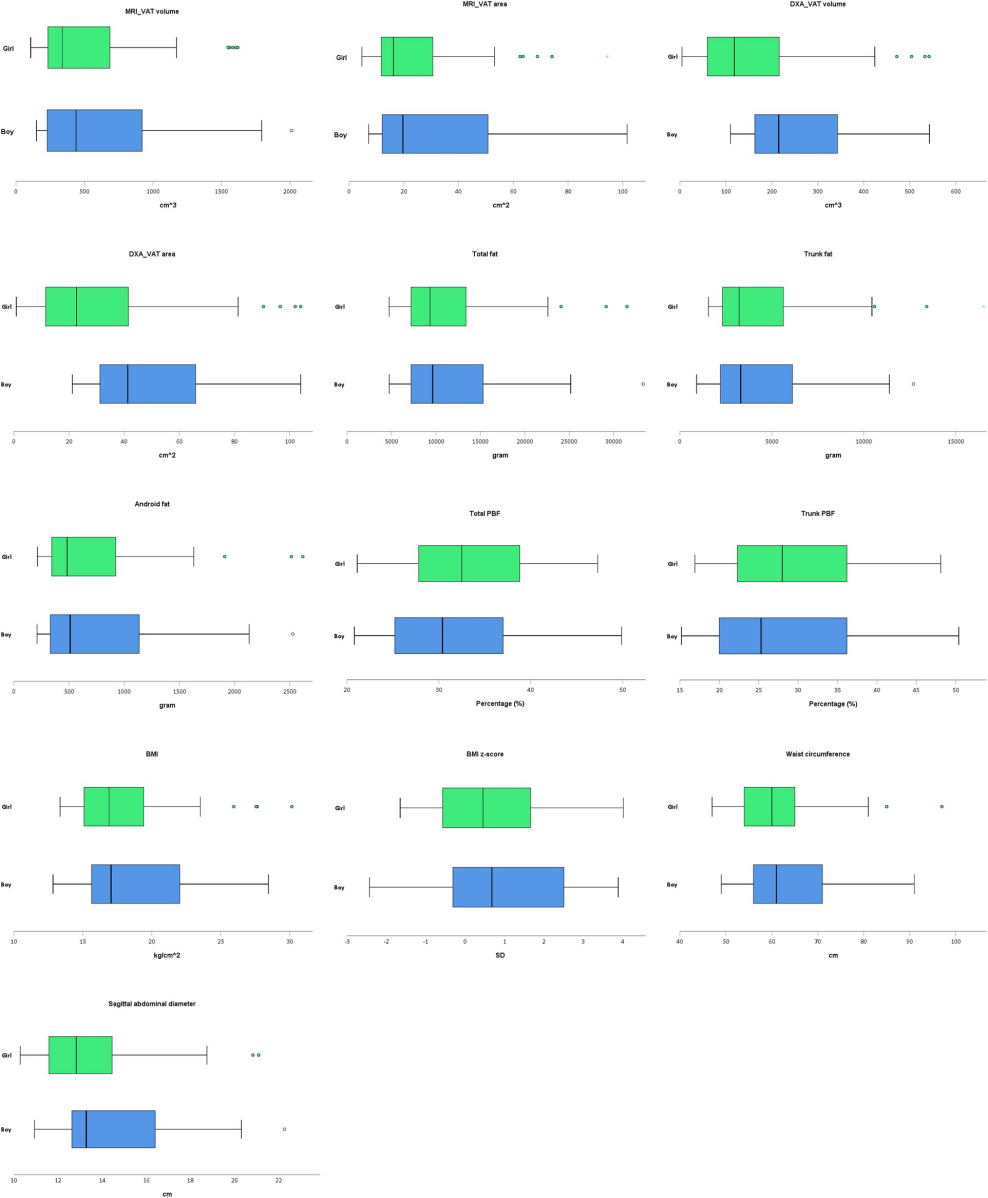
Visceral fat estimates among the study population. In the boxplot, the thick line in the middle of the box is the median. The left and right edges of the box are located at the first and third quartiles. The whiskers show the maximum and minimum values, with the exceptions of outliers (circles) and extremes (asterisks).

A total of 112 children aged 7–10 years were enrolled in the study; of these, one was excluded due to refusal to take venous sampling, and thus 111 individuals comprised the final study sample. Of the 111 children, 57 were boys (51.4%). The average age was 8.6 years and the mean BMI z-score was 0.8 (range: -2.4 to 4.0). Subject characteristics are shown in **Table 1** and **Fig 1**. Boys were significantly older, taller and had greater VAT_DXA_ than girls. For the other variables, there were no statistically significant differences between the sexes. Children were considered as “at risk” of cardiometabolic components, according to the methodology section. In boys, there were 14, 27 and 24 subjects with diabetes, lipid and hypertensive risks, respectively. There were 20 boys with at least two risks. In girls, there were 5, 23 and 25 subjects with diabetes, lipid and hypertensive risks, respectively. There were 18 girls with at least two risks. In both sexes, the prevalence of lipid and hypertensive risk components were higher than that of diabetes risk.

**Table 1:** Descriptive characteristics of children.

Variables	Boys (n = 57)		Girls (n = 54)		p value
	Mean	SD	Mean	SD	
**Basic indices**					
Age (yr)	8.8	1.1	8.3	1.1	0.046
Height (cm)	135.6	8.0	131.9	8.6	0.021
Weight (kg)	35.3	10.9	31.8	10.6	0.091
BMI (kg/m^2^)	18.9	4.1	17.9	3.9	0.198
BMI z-score	1.0	1.6	0.6	1.4	0.175
WC (cm)	64.0	10.5	61.0	9.7	0.120
**Cardiometabolic risk variables**					
FBG	92.3	6.2	90.2	5.5	0.069
HbA_1c_ (%)	5.4	0.2	5.3	0.3	0.130
HOMA-IR	1.4	0.9	1.6	1.4	0.408
TC (mg/dl)	167.4	22.3	166.4	23.8	0.879
TG (mg/dL)	68.4	28.4	62.1	28.6	0.251
HDL (mg/dL)	60.1	13.5	59.8	11.7	0.886
LDL (mg/dL)	93.3	22.1	94.2	20.2	0.815
Non-HDL-C (mg/dl)	107.0	23.6	106.6	21.5	0.941
SBP (mmHg)	108.3	9.8	104.8	11.5	0.090
DBP (mmHg)	61.5	7.9	62.8	8.8	0.424

**Abbreviation:** SD: standard deviation; BMI: body mass index; WC: waist circumference; FBG: fasting blood glucose HbA_1c_: glycosylated haemoglobin; HOMA-IR: homeostatic model assessment for insulin resistance; TC: total cholesterol; TG: triglycerides; HDL: high-density lipoprotein; LDL: low-density lipoprotein; non-HDL-C: non high-density lipoprotein-cholesterol; SBP: systolic blood pressure; DBP: diastolic blood pressure.

**Tables 2 and 3** show the correlation coefficients between VAT methods in boys and girls, respectively. All the test VAT methods were positive correlated with VAT_MRI_ volume with high to perfect correlations (r ≥ 0.790). It is generally agreed that VAT_MRI_ volume is the reference measure for VAT. In our study, the single-slice VAT area obtained at the L4 level were shown to highly correlate with VAT_MRI_ volume in both sexes, with a correlation coefficient of r = 0.930 for boys and r = 0.843 for girls, indicating that single-slice VAT_MRI_ area is a good surrogate for VAT_MRI_ volume. The correlation between VAT_MRI_ and the other VAT methods was higher in boys than in girls, except for SAD. All VAT methods were better correlated with VAT_MRI_ volume than area. DXA estimates were better correlated with VAT_MRI_ volume than basic indices, except for PBF and PBF_TR. It is also worth noting that BMI was better correlated with VAT_MRI_ than BMI z-score. As expected, perfect positive correlations (r = 1.000) were found between VAT_DXA_ area and volume in both sexes, indicating that the values of VAT_DXA_ volume were derived from those of VAT_DXA_ area. The correlation coefficients between VAT_DXA_ and VAT_MRI_ volume were 0.954 for boys and 0.951 for girls.

**Table 2:** Pearson correlation between VAT_MRI_ and other predictors in boys.

Correlation	VAT_MRI_ (cm^3^)	VAT_MRI_ (cm^2^)	VAT_DXA_ (cm^3^)	VAT_DXA_ (cm^2^)	Total fat	Trunk fat	Android fat	Total PBF	Trunk PBF	BMI	BAZ	WC	SAD
VAT_MRI_ (cm^3^)	1.000	0.930	0.954	0.954	0.972	0.971	0.983	0.895	0.934	0.934	0.820	0.902	0.918
VAT_MRI_ (cm^2^)		1.000	0.912	0.912	0.894	0.890	0.912	0.854	0.884	0.883	0.802	0.824	0.905
VAT_DXA_ (cm^3^)			1.000	1.000	0.948	0.936	0.955	0.933	0.951	0.951	0.888	0.915	0.892
VAT_DXA_ (cm^2^)				1.000	0.948	0.936	0.955	0.933	0.951	0.951	0.888	0.915	0.892
Total fat					1.000	0.986	0.983	0.920	0.942	0.959	0.839	0.918	0.933
Trunk fat						1.000	0.982	0.884	0.921	0.930	0.791	0.892	0.918
Android fat							1.000	0.910	0.946	0.947	0.831	0.908	0.923
PBF								1.000	0.986	0.930	0.905	0.848	0.839
PBF_TR									1.000	0.940	0.888	0.865	0.864
BMI										1.000	0.948	0.936	0.930
BAZ											1.000	0.853	0.822
WC												1.000	0.885
SAD													1.000

**Abbreviation:** VAT_MRI_: visceral adipose tissue estimates by magnetic resonance imaging; VAT_DXA_: visceral adipose tissue estimates by dual energy X-ray absorptiometry; BPF: percent body fat; PBF_TR: percent body fat at the trunk; BMI: body mass index BAZ: BMI z-score; WC: waist circumference; SAD: sagittal abdominal diameter.

**Table 3:** Pearson correlation between VAT_MRI_ and other predictors in girls.

Correlation	VAT_MRI_ (cm^3^)	VAT_MRI_ (cm^2^)	VAT_DXA_ (cm^3^)	VAT_DXA_ (cm^2^)	Total fat	Trunk fat	Android fat	PBF	PBF_TR	BMI	BAZ	WC	SAD
VAT_MRI_ (cm^3^)	1.000	0.843	0.951	0.951	0.942	0.939	0.939	0.790	0.830	0.915	0.814	0.892	0.918
VAT_MRI_ (cm^2^)		1.000	0.849	0.849	0.745	0.759	0.752	0.676	0.736	0.761	0.715	0.702	0.828
VAT_DXA_ (cm^3^)			1.000	1.000	0.947	0.957	0.956	0.859	0.901	0.950	0.884	0.888	0.932
VAT_DXA_ (cm^2^)				1.000	0.947	0.957	0.956	0.859	0.901	0.950	0.884	0.888	0.932
Total fat					1.000	0.990	0.985	0.855	0.878	0.965	0.856	0.943	0.943
Trunk fat						1.000	0.991	0.840	0.879	0.961	0.840	0.918	0.938
Android fat							1.000	0.828	0.864	0.960	0.839	0.929	0.934
PBF								1.000	0.982	0.852	0.873	0.796	0.787
PBF_TR									1.000	0.875	0.873	0.816	0.832
BMI										1.000	0.937	0.914	0.947
BAZ											1.000	0.807	0.858
WC												1.000	0.906
SAD													1.000

Sensitivity and specificity are common metrics for quantifying the diagnostic accuracy of a test. A ROC curve plots the sensitivity against 1-specificity for all possible cut-off values and the AUC is a scalar measure of the overall performance of diagnostic tests with continuing variables. To assess of performance of VAT tools for different genders and cardiometabolic risks, subjects were stratified by sex and risk factors for ROC analysis. The AUC of different VAT tools for the diagnosis of a range of cardiometabolic risk factors in boys and girls are shown in **Table 4** and **Table 5**, respectively. For the diagnosis of lipid risk, all VAT methods had acceptable to excellent performance in boys (AUC = 0.753–0.837), whereas all methods yielded poor performance in girls, except for VAT_MRI_ volume (AUC = 0.722). For the diagnosis of hypertensive risk, all VAT methods had acceptable to excellent performance in boys (AUC = 0.710–0.821), but poor or no discrimination ability in girls. In both sexes, all VAT methods had no or poor discrimination ability for diabetes risk. For the diagnosis of at least two obesity-related risks, all VAT methods had excellent to outstanding discriminating ability in boys (AUC = 0.861–0.945), but no or poor discrimination ability in girls.

**Table 4:** ROC analysis (boys).

	Diabetes risk			Lipid risk			Hypertensive risk			≥ 2 risks		
	AUC	Std. Error	p	AUC	Std. Error	p	AUC	Std. Error	p	AUC	Std. Error	p
VAT_MRI_ (cm^3^)	0.638	0.093	0.124	0.812	0.061	<0.001	0.789	0.063	<0.001	0.934	0.036	<0.001
VAT_MRI_ (cm^2^)	0.669	0.080	0.059	0.753	0.069	0.001	0.773	0.068	<0.001	0.895	0.047	<0.001
VAT_DXA_ (cm^2^)	0.652	0.085	0.090	0.790	0.063	<0.001	0.812	0.058	<0.001	0.932	0.033	<0.001
Total fat	0.653	0.093	0.088	0.827	0.055	<0.001	0.809	0.059	<0.001	0.943	0.029	<0.001
Trunk fat	0.656	0.089	0.081	0.837	0.055	<0.001	0.821	0.055	<0.001	0.945	0.030	<0.001
Android fat	0.657	0.091	0.080	0.836	0.055	<0.001	0.799	0.061	<0.001	0.944	0.031	<0.001
PBF	0.698	0.091	0.027	0.805	0.063	<0.001	0.803	0.060	<0.001	0.941	0.029	<0.001
PBF_TR	0.688	0.094	0.036	0.816	0.062	<0.001	0.801	0.060	<0.001	0.943	0.030	<0.001
BMI	0.643	0.087	0.111	0.800	0.063	<0.001	0.812	0.058	<0.001	0.932	0.034	<0.001
BMI z-score	0.640	0.085	0.117	0.802	0.063	<0.001	0.793	0.061	<0.001	0.927	0.035	<0.001
WC	0.569	0.101	0.442	0.802	0.060	<0.001	0.788	0.065	<0.001	0.886	0.055	<0.001
SAD at L4	0.614	0.090	0.204	0.778	0.064	<0.001	0.710	0.080	0.007	0.861	0.059	<0.001

**Table 5:** ROC analysis (girls).

	Diabetes risk			Lipid risk			Hypertensive risk			≥ 2 risks		
	AUC	Std. Error	p	AUC	Std. Error	p	AUC	Std. Error	p	AUC	Std. Error	p
VAT_MRI_ (cm^3^)	0.596	0.137	0.483	0.722	0.072	0.006	0.598	0.079	0.218	0.617	0.086	0.163
VAT_MRI_ (cm^2^)	0.604	0.115	0.447	0.661	0.078	0.045	0.617	0.079	0.143	0.599	0.09	0.24
VAT_DXA_ (cm^2^)	0.567	0.145	0.622	0.683	0.075	0.022	0.632	0.076	0.098	0.599	0.087	0.24
Total fat	0.588	0.124	0.521	0.682	0.076	0.023	0.61	0.077	0.168	0.596	0.085	0.255
Trunk fat	0.596	0.117	0.483	0.67	0.077	0.034	0.601	0.078	0.202	0.582	0.086	0.331
Android fat	0.580	0.133	0.561	0.682	0.075	0.023	0.632	0.075	0.098	0.600	0.085	0.233
PBF	0.563	0.090	0.644	0.642	0.078	0.077	0.574	0.080	0.353	0.549	0.087	0.557
PBF_TR	0.578	0.102	0.571	0.636	0.078	0.090	0.572	0.079	0.362	0.534	0.088	0.686
BMI	0.612	0.102	0.412	0.651	0.078	0.060	0.666	0.073	0.037	0.660	0.077	0.056
BMI z-score	0.614	0.087	0.403	0.646	0.078	0.069	0.668	0.073	0.034	0.684	0.073	0.028
WC	0.502	0.143	0.988	0.673	0.075	0.031	0.644	0.075	0.070	0.577	0.085	0.359
SAD at L4	0.496	0.134	0.976	0.643	0.08	0.074	0.628	0.077	0.109	0.596	0.085	0.255

## Discussion

In this study, DXA-derived VAT area and volume were very highly correlated with MRI-derived VAT, indicating that VAT_DXA_ can act as a predictor of VAT in healthy pre-pubertal children. In addition, all other VAT measures and total VAT_MRI_ volume were high to perfectly correlated in pre-pubertal children, indicating a very strong tendency for these methods to estimate VAT. The two VAT methods that had the highest correlation coefficient were VAT_DXA_ area and VAT_DXA_ volume (r = 1.000), suggesting the values of VAT_DXA_ volume were derived from the VAT_DXA_ area. Further, using the VAT_DXA_ value to predict cardiometabolic risks was explored. In contrast to what previous work has shown, we found that VAT area and volume by DXA were no better at predicting VAT_MRI_ and cardiometabolic risks than the standard DXA-derived total body, trunk fat and android fat masses. Furthermore, VAT measures helped distinguish lipid risk, hypertensive risk and at least two cardiometabolic risk factors in boys, but not in girls. However, our results suggested no or poor discrimination ability to diagnose diabetes risk in children using VAT estimates in both sexes.

Volumetric CT and MRI measurements are the gold standard methods for VAT quantification *in vivo*. However, single-slice VAT area is more commonly used in body composition research than the VAT volume, due to the labour-intensive and time-consuming process of image segmentation. Validation of single-sliced VAT area has been done previously. In adults, the single-slice VAT area showed high to very high correlation with total VAT volume, with the highest correlation in slices taken at 5–10 cm above L4-L5 in healthy adults (r = 0.97–0.99) [29–32]. The correlations between single-slice VAT area and total VAT volume was also high to very high in school-aged children (r ≥ 0.874), but the best single-slice location to represent total VAT volume varied between studies [33–35]. It is not clear why the best single-slice location for VAT differs between children and adults. One possible explanation is that patterns of adiposity may vary with age. Consistent with previous studies, our study showed that single-slice MRI at the L4 level was highly to very highly correlated with VAT_MRI_ volume in healthy children (r = 0.930 in boys and r = 0.843 in girls).

As far as we know, only two studies have sought to validate VAT_DXA_ against CT or MRI in children; both studies were performed using Lunar scanners and neither took gender differences into account [20, 21]. Lee et al. [21] showed that VAT_DXA_ was highly correlated with VAT_MRI_ at multiple lumbar disc levels in 32 girls aged 9–13 years (r = 0.70–0.79). In another study, Bosch et al. [20] reported that the correlations between VAT_DXA_ and VAT_CT_ were 0.55 and 0.63 for children aged 6–11 years and 12–18 years, respectively. In that study, the correlation was high for children with BMI above the 85^th^ percentile (r = 0.859), but negligible in children with lower BMI (r = 0.226). Indeed, Lunar DXA scanners provided much lower correlation coefficients between VAT_DXA_ and standard methods in children than those of adults in previous studies (r > 0.9) [14, 15, 19]. Using a Hologic scanner, our results were consistent with previous studies, showing very high correlations between VAT_DXA_ and VAT_MRI_ in children with a wide range of BMI (r = 0.954 in boys and r = 0.951 in girls). The estimation models of VAT_DXA_ include similar variables and perform well in adults for both Hologic and Lunar scanners [14, 15]. It is not clear why the performance differed between two DXA models in children.

This study analysed gender differences for predicting cardiometabolic risks using VAT methods. According to AUC results, VAT methods were useful in accurately stratifying cardiometabolic risks for boys but not for girls. Body composition and cardiometabolic risks are affected by sex hormones and it is well known that gender differences present in discriminating cardiometabolic risks using VAT in adults [31, 36, 37]. In children, there is sexual dimorphism in fat distribution and the distribution is present in early life but emerges more dramatically during puberty [3, 38]. Our study suggested that gender differences in the ability to differentiate cardiometabolic risks using VAT were already present in prepubertal children.

The ability of a range of VAT methods to predict cardiometabolic risks was investigated in the present study. For the diagnosis of hypertensive risks, all methods showed acceptable to excellent performance in boys, but sub-optimal performance in girls. The issue of gender differences in the prediction of hypertensive risk using VAT has been observed in previous studies, but with inconsistent results. Daniels et al. [39] reported a significant positive relationship between android fat by DXA and hypertensive risk in 127 healthy children aged 9–17 years, but the study did not account for sex differences. Zhao et al. [40] reported a significant correlation between WC and hypertensive risk in Chinese children aged 7–16 years (n = 1626), regardless of sex. Similar to our results, He et al. [41] reported that the total fat and trunk fat as measured by DXA were predictors of blood pressure in boys at all pubertal stages, but not in girls, in 920 healthy children aged 5–18 years. Syme et al. [42] also reported that the positive association between VAT_MRI_ and blood pressure was less in girls compared to that in boys in healthy adolescents (n = 425). In this study, all VAT methods had acceptable to excellent performance for the accurate diagnosis of lipid risk in boys, whereas only VAT_MRI_ had an acceptable diagnostic performance in girls. Previous studies have reported associations between VAT and lipid risk in children, but the authors did not report gender differences [34, 39]. Daniels et al. [39] showed that android fat by DXA was associated with lipid risk in children aged 9–17 years, whereas Lee et al. [34] demonstrated that single-slice VAT_MRI_ areas could be used to predict lipid risk. For the diagnosis of diabetes risks, all VAT methods in this study showed sub-optimal performance, regardless of sex, suggesting that VAT may not be appropriate to identify pre-pubertal children at risk of diabetes. Our results contradicted those of previous studies which showed a correlation of VAT with diabetes risks in children [20, 43–45]. Although the reason for this discrepancy is unclear, it might be caused by the younger age of the subjects in our study.

The fundamental differences between DXA-derived total fat, trunk fat and android fat versus DXA-derived VAT lie in their different methodologies of calculating fat masses. DXA-derived total fat, trunk fat and android fat are based on solving the attenuation equations obtained at two energy levels of X-ray [7]. In contrast, the VAT_DXA_ area is calculated as the total fat minus the subcutaneous fat, where subcutaneous fat is derived from regression analysis, with the transverse diameter and the inner abdominal muscle wall diameter as independent variables [14, 15]. In spite of the methodological differences, our study showed that these estimates were all very highly correlated with VAT_MRI_ volume and their prediction abilities for obesity-related risks were compatible in pre-pubertal children. Simple anthropometric indices are widely used measures, but our study showed that these indices were highly to very highly correlated with VAT_MRI_, and their ability to discriminate obesity-related risks were compatible to VAT_MRI_, indicating that they are also very useful VAT indicators.

This study had the strengths of a homogeneous subject population in terms of ethnicity and pubertal stage, but a wide range of body fatness, as well as providing discrimination abilities for cardiometabolic risks among common VAT methods. However, our study has certain methodological limitations. First, because this is a cross-sectional study, we could not show causality between VAT and risk factors. Second, we did not use the standard definitions of abnormal values for cardiometabolic risks, as there is currently no universal agreement on the definition of childhood metabolic risk factors for pre-pubertal children. Third, the results of this study were limited to pre-pubertal children aged 7–10 years using a Hologic scanner, and might not be applicable to children at a different age group or studies using a Lunar DXA scanner.

Accurate VAT assessment is challenging. This study demonstrated that VAT estimates derived from the new generation of DXA scanners were very highly correlated with current standard methods, indicating that VAT_DXA_ can be an accurate VAT predictor in healthy pre-pubertal children. However, the discrimination abilities for cardiometabolic risks were equivalent to conventional DXA-derived fat estimates, indicating that VAT_DXA_ may not be a more robust VAT surrogate than the existing DXA-derived fat estimates such as total fat, trunk fat and android fat.

## Supporting information

S1 Fig (PPTX)

S1 Dataset (CSV)
