# Supplementary material for: Added values of DXA-derived visceral adipose tissue to discriminate cardiometabolic risks in pre-pubertal children
Source: PLoS One. 2020 May 13;15(5):e0233053. doi: 10.1371/journal.pone.0233053 (PMC7219764; doi:10.1371/journal.pone.0233053)
Supplement: S1 Fig — (PPTX) [file pone.0233053.s001.pptx]

## Slide 1
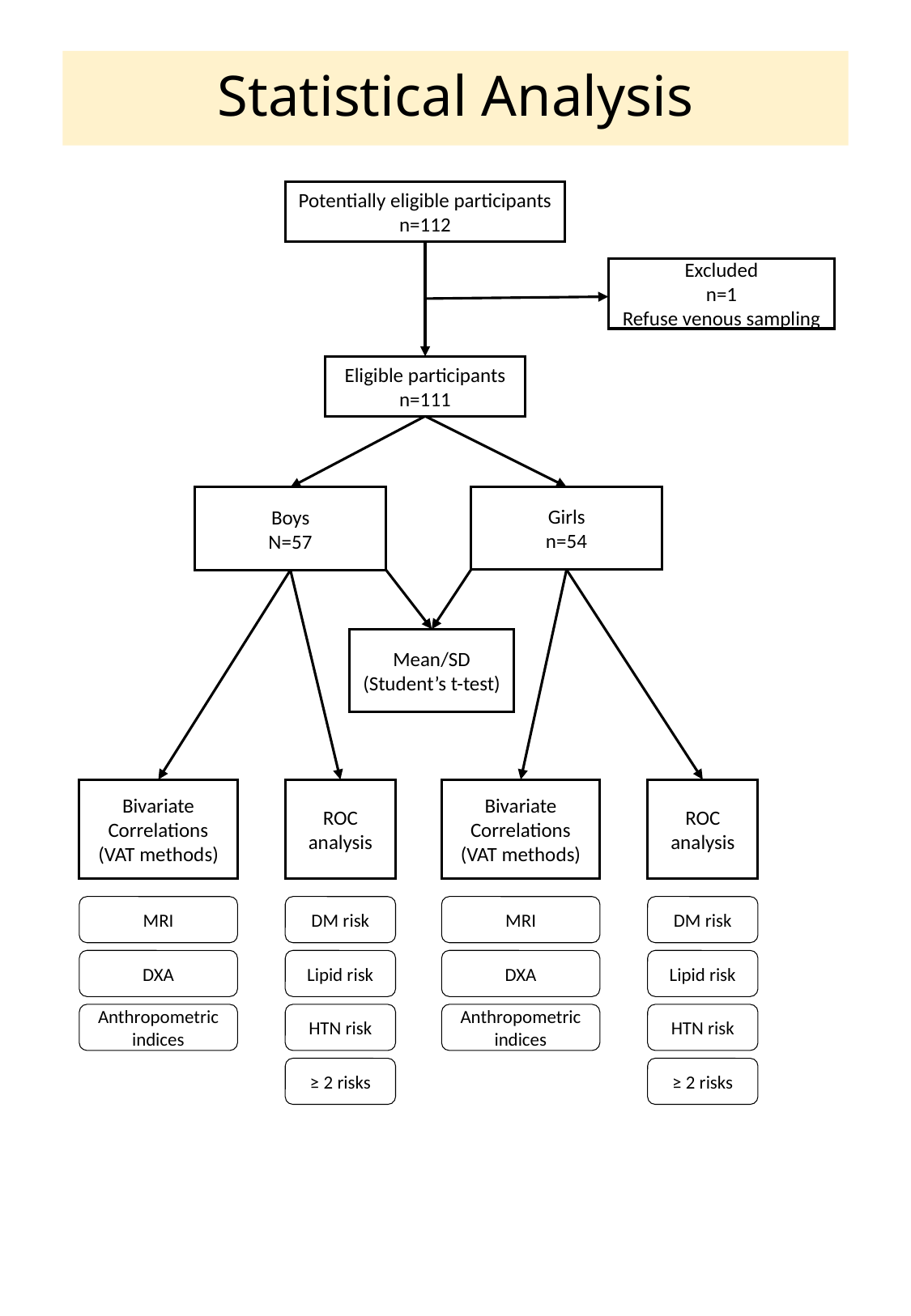

# Statistical Analysis
Potentially eligible participants
n=112
Excluded
n=1
Refuse venous sampling
Eligible participants
n=111
Girls
n=54
Boys
N=57
Mean/SD
(Student’s t-test)
Bivariate
Correlations
(VAT methods)
ROC analysis
Bivariate
Correlations
(VAT methods)
ROC analysis
MRI
DM risk
MRI
DM risk
DXA
Lipid risk
DXA
Lipid risk
Anthropometric indices
HTN risk
Anthropometric indices
HTN risk
≥ 2 risks
≥ 2 risks
